# Towards a Remote Monitoring of Patient Vital Signs Based on IoT-Based Blockchain Integrity Management Platforms in Smart Hospitals

**DOI:** 10.3390/s20082195

**Published:** 2020-04-13

**Authors:** Faisal Jamil, Shabir Ahmad, Naeem Iqbal, Do-Hyeun Kim

**Affiliations:** Department of Computer Engineering, Jeju National University, Jejusi 63243, Korea; faisal@jejunu.ac.kr (F.J.); shabir@jejunu.ac.kr (S.A.); naeemiqbal@jejunu.ac.kr (N.I.)

**Keywords:** Internet of things, smart contract, hyperledger fabric, blockchain, smart healthcare

## Abstract

Over the past several years, many healthcare applications have been developed to enhance the healthcare industry. Recent advancements in information technology and blockchain technology have revolutionized electronic healthcare research and industry. The innovation of miniaturized healthcare sensors for monitoring patient vital signs has improved and secured the human healthcare system. The increase in portable health devices has enhanced the quality of health-monitoring status both at an activity/fitness level for self-health tracking and at a medical level, providing more data to clinicians with potential for earlier diagnosis and guidance of treatment. When sharing personal medical information, data security and comfort are essential requirements for interaction with and collection of electronic medical records. However, it is hard for current systems to meet these requirements because they have inconsistent security policies and access control structures. The new solutions should be directed towards improving data access, and should be managed by the government in terms of privacy and security requirements to ensure the reliability of data for medical purposes. Blockchain paves the way for a revolution in the traditional pharmaceutical industry and benefits from unique features such as privacy and transparency of data. In this paper, we propose a novel platform for monitoring patient vital signs using smart contracts based on blockchain. The proposed system is designed and developed using hyperledger fabric, which is an enterprise-distributed ledger framework for developing blockchain-based applications. This approach provides several benefits to the patients, such as an extensive, immutable history log, and global access to medical information from anywhere at any time. The Libelium e-Health toolkit is used to acquire physiological data. The performance of the designed and developed system is evaluated in terms of transaction per second, transaction latency, and resource utilization using a standard benchmark tool known as Hyperledger Caliper. It is found that the proposed system outperforms the traditional health care system for monitoring patient data.

## 1. Introduction

Among fundamental human rights, one of the foremost is the availability of health facilities [1]. Different health facilities such as hospitals, healthcare, and pharmacies are well equipped with resources to provide health services to humans. Advancement in the field of information and communication technology (ICT) has paved a way towards connecting physical devices with wearable sensors. The use of software and electronic devices allows the collection, processing, and generation of data over a large-scale network in an efficient and effective way. Despite the abundance of health facilities, fatal diseases such as heart disease, cancer, influenza, and pneumonia have grown significantly and take many human lives. A large number of doctors, therapists, nurses, and other staff constantly monitor and observe the health of patients. Patients with chronic diseases are monitored and observed regularly. Over the past few years, different healthcare monitoring systems have been introduced, employed to collect, process, and analyze data retrieved from sensing devices [1,2,3,4]. These healthcare systems are also responsible for monitoring and observing patient vital signs. However, the issue of legal interoperability arises when connecting different departments of a hospital to share medical data effectively to provide better healthcare services to patients. There is no centralized system for medical data management and sharing that improves the effective delivery of healthcare services to patients.

Electronic Medical Record (EMR) data, notably Protected Health Information (PHI), suffers from high probability risk. There have been many cases reported in the past few years that are related to the unauthorized exposure and leaking of personal healthcare data [5]. In the US and other European countries, medical data is protected using privacy protection regulation and governance, e.g., the Health Insurance Portability and Accountability Act (HIPAA) [6,7] which requires storing and sharing medical data in a secure and protected way. Therefore, to enhance data privacy and security, healthcare providers, e.g., hospitals, clinics, and pharmacies, have decided to develop a closed private network. The closed private network contains parameters such as firewalls and intrusion detection systems (IDS). Similarly, medical data is increasing at the rate of 20%–40% per year, and an average of 665 terabytes of medical information was managed in 2015 by US health providers. It is estimated that by 2020 healthcare data will have reached an average of 25,000 petabytes, which is extremely challenging to manage and process in a private local network domain [5].

Presently, cross-institutional data interoperability is one of the main issues faced by both patients and healthcare providers in the healthcare ecosystem. Due to the large scale of data generated every day, it is almost impossible to process, analyze, and store data on a local domain. Therefore, many healthcare providers have shifted their data to a public domain. Still, the problem is a lack of interoperability regarding medical information, which further poses a threat to medical analytics that require a large amount of medical information. Furthermore, it creates a barrier for patients seeking treatment, as their data is scattered across multiple places, e.g., hospital, pharmacy, clinic, etc. Therefore, a more integrated and holistic healthcare infrastructure is needed to enable the interoperability and secure sharing of medical data among various healthcare domains to boost the collaborative healthcare service and research.

However, personal medical systems consist of essential requirements, such as data sharing, data security and consistency, data reliability, and convenience [8]. These requirements of personal medical data are the most important for interaction with and collection of EMR. Traditional healthcare systems are not able to meet these crucial requirements for an efficient process because they have no consistent and reliable structure in terms of data security, sharing, and access control models. Therefore, it is necessary to have a new secured system to enhance the data-access process under the privacy and security of government regulations to ensure accountability and monitoring of medical usage data [9]. Blockchain is a secure and transparent distributed ledger, and it paves the way for a revolution in existing healthcare systems by integrating its unique features.

Blockchain is essentially a distributed ledger (database) that can be programmed to record online financial transactions in such a way that they are secure and cannot be manipulated. In the blockchain, each transaction is digitally signed from participants to ensure its authenticity and security. The distributed ledger operates by consensus (smart contracts). Both parties agree at the ledger to put each transaction into a block and validate that block to add it to a chain. Each block contains data and information. Finally, that chain is protected using cryptography algorithms, and consequently, it cannot be manipulated or changed. Blockchain is a decentralized technology that ensures the security of data, and no one can manipulate transaction data because of its many replicas in different servers. Data resources stored in centralized servers are vulnerable to cybercrime. On the other hand, blockchain ensures the security and privacy of data by storing it in decentralized locations [10,11]. Over the past few years, the market value of blockchain technology increased substantially. Blockchain technology is more trusted and secure than all other record-keeping systems. It is a distributed ledger where all nodes of the network share the same documentation. It is also used to increase efficiency and speed by automating the traditional process with blockchain technology. It also reduces costs because it does not require the buying and selling of products through third parties.

The proposed healthcare IoT brings the following contribution to the state of the art related IoT:

- Scalability: Our proposed solution meets the requirements of numerous healthcare IoT devices connected in a practical IoT network through different constrained networks to a single blockchain.
- Throughput: We propose a blockchain-based private network to enable the interaction of only those entities that are authenticated or registered, which will lead to improving the throughput of the network. In this paper, we use permissioned blockchain to enable interaction among a set of network nodes fully trusting each other. Therefore, the conventional protocols, such as crash fault-tolerant (CFT) and byzantine fault-tolerant (BFT), can be used to enhance the throughput of the network.
- Transparency: The proposed system is transparent because it hides the information of IoT devices and the transaction log from unauthorized users.
- Lightweight: The proposed system uses a RESTful API, which allows cross-platform communication between IoT devices and the blockchain network. The inclusion of RESTful API offloads the computation that has needed to be done on the blockchain network in the past.

In this paper, we propose a novel medical blockchain model for the secure monitoring of patient vital signs in smart hospitals. Our proposed system is based on smart hospitals where patients are equipped with healthcare devices in such a way that these devices read the vital signs of patients and share them with other authorized users in the blockchain network. The proposed medical platform stores personal healthcare data on a secured permission chain of the network. The designed system is based on a web-driven paradigm with the development of web front-end technology, i.e., HTML5 and JavaScript, in order to improve resource management within the network. Similarly, the blockchain also provides product-centric services through Representational State Transfer Application Programming Interfaces (REST API), which are triggered either through IoT devices or a web client. The healthcare IoT devices can be controlled by the authorized user of the system. These devices are aware of changes in context so that they can adapt themselves to those changes. For instance, whenever the value of a sensor reaches an abnormal value, the system issues an alert notification to acknowledge the change. Moreover, for experimental analysis, we have used the Libelium e-health Biometric Sensor Platform toolkit [12] to acquire physiological data, e.g., EEG, ECG, EMG, and blood pressure, etc. Furthermore, we have created the REST API, which is used to expose application-specific services provided by the secured and distributed network. A smart contract provides controlled access to the ledger to ensure data confidentiality and consistency of patient vital-sign information and to host the ledger functions across the network. Moreover, the access control policy is implemented to authorize participants and users of the system to access authorized content and transactions, e.g., only a doctor is allowed to access and manipulate the IoT device. As blockchain technology is not capable of storing large transaction data payloads, there is a need for a storage technology that can store the massive amount of healthcare sensor data. In the design system, we used the couch database instead of using a traditional database to avoid data redundancy and intended to store large transactions of data. Hence, the couch database is deployed on each peer to provide file storage across the entire blockchain system. Furthermore, we used a benchmark tool known as Hyperledger Caliper [13] to evaluate the performance of the developed system in terms of TPR, TL, and RT. Finally, we prove the practicability of the developed system by designing and implementing a real-life case study in a smart space. The study was conducted in accordance with the Declaration of Helsinki, and the protocol was approved by the Ethics Committee of Mobile Computing Lab, Jeju National University, South Korea (Project Identification code: D423).

The remainder of this paper is organized as follows: Section 2 gives an introduction to healthcare vital signs and gives details on related projects and their limitations. Section 3 explains the medical blockchain scenario for secure monitoring of patient vital signs in smart hospitals, the transaction process of the proposed medical blockchain platform, and the smart contract design. Section 4 presents the implementation of the proposed blockchain-based system. Section 5 presents the development environment of the proposed blockchain-based system. Section 6 presents a performance evaluation of the designed and developed medical platform using blockchain technology and highlights the designed platform through a benchmark analysis with existing work and also through Hyperledger Caliper. Section 7 concludes the paper with future research directions.

## 2. Literature Review

**Figure 1:**
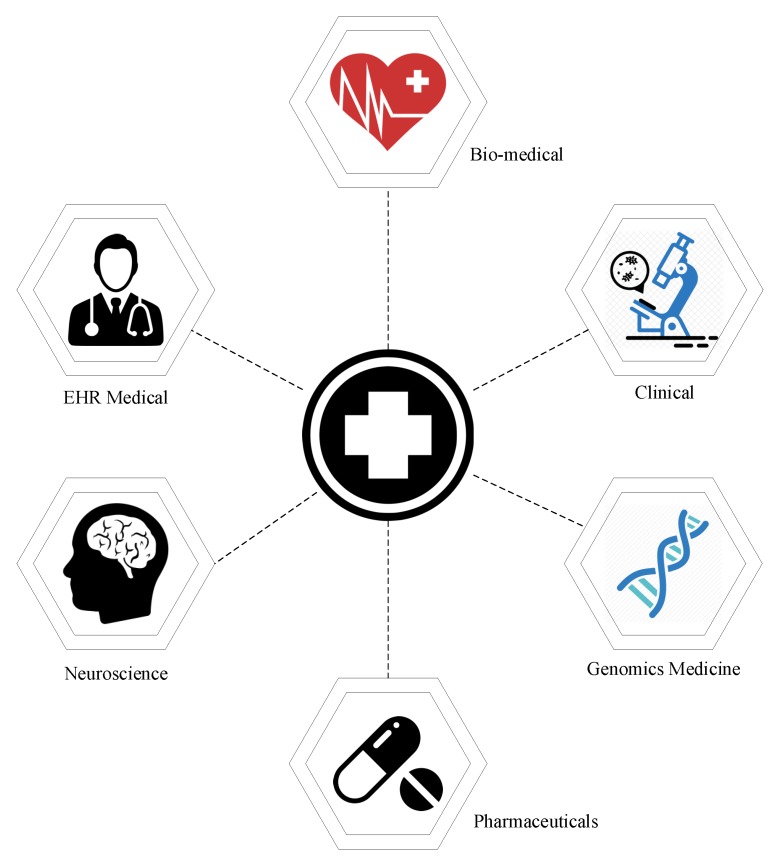
Applications of blockchain in healthcare.

Healthcare applications serve many purposes, such as decision-making, workflows, and clinical facts, electronic health records (EHR), genomics medicine, neuroscience, biomedical, and pharmaceuticals. Data standardization and communication protocols can enable IoT technologies to deliver efficient healthcare services. Better connectivity, user interfaces, the security of patient data, and data interoperability can reduce the challenges of providing efficient healthcare services. Presently, healthcare is one of the most popular ongoing research domains; researchers strive to make more reliable healthcare applications for the community and healthcare industry. Several stakeholders, such as patients, hospitals, and pharmacies, need to maintain, share, and access health records in a secure way without any changes. Blockchain is the emerging technology in the current era and has the potential to address the challenges of the healthcare industry. The following are some of the blockchain-based healthcare applications, as illustrated in Figure 1.

### 2.1. Blockchain in EHR

Over the past few years, many systems have helped in digitizing, sharing, and offering easy access to medical records to both patients and hospitals. In this section, we discuss a few blockchain-based EHR systems.

MedRec is the healthcare platform that provides decentralized record management, data sharing, and authorization among different users of the system. Patients can store, manage, and also authorize other stakeholders to access their records. The designed MedRec framework can store medical records on the blockchain database using a key-value format instead of a pointer to a data storage location [14,15,16]. Blockchain-based healthcare platform Gem is designed and developed by Gem Health Network using Ethereum blockchain. The implemented system addresses the operational cost of data management. The proposed system also provides interoperability among various stakeholders to access the same information to maintain better healthcare services [17]. Presently, researchers use patient healthcare information for research studies based on healthcare organizations. Healthbank is a platform that enables every patient to store and manage their medical data and provides healthcare data to researchers and pharmaceutical companies. The designed Healthbank system also provides incentives to patients for their contribution to the system [17,18]. Blockchain-Based Data Sharing (BBDS) is a permissioned blockchain platform that enables secure, scalable data transaction encrypted with a cryptographic algorithm. The HDG (Healthcare Data Gateway) is a smartphone application gateway that is built over a blockchain-enabled cloud [19]. In [20] is a permissioned blockchain is built using the Ethereum platform to provide a secure and safe remote patient-monitoring system. The designed platform is a secure real-time monitoring system that allows the stakeholder to participate and track their health records, and also provides remote patient monitoring. The system maintains a secure, safe, and up-to-date patient history. Ivan, in [21], presented a secure health data storage system developed using a decentralized database based on blockchain technology. The system has the capability to store encrypted healthcare data publicly and use the system like a blockchain-based personal health record (PHR). Moreover, the PHR system also enables the patient not only to access and monitor their data but also allow the patient to share the data with other healthcare personnel. The authors in [22] presented a blockchain-based remote treatment and diagnosis of cancer patients. The system uses a smart contract to ensure the validity and security of patient health information. A telemonitoring system handles securely shared specialized patient healthcare data at different healthcare centers. Mannaro et al. in [22] proposed a blockchain-based online dermatology system for assisting patients suffering from skin diseases. Similarly, the authors in [23] proposed a blockchain-based ProActive Aging system that provides support for aging people. The system is capable of monitoring patient chronic diseases, e.g., cancer, etc. Blockchain is an ideal technology for remote patient monitoring and support. MediBloc [24] is a blockchain-based EMR platform based on the decentralized open-source protocol used to store healthcare data for healthcare providers, researchers, and patients. The application is developed based on the Ethereum Virtual Machine (EVM), which uses a points-based system to evaluate user participation. Afterwards, the coin token uses medical transaction as an insurance payment. Healthcoin [25] is a blockchain-based permission-less currency which is used to verify healthcare transactions.

### 2.2. Blockchain in Clinical Research

Clinical trials are a medical approach used to prevent and diagnose disease. During recent decades, many systems have been developed to avoid and diagnose diseases. Still, these systems have some issues, for example, data integrity, record-sharing, data privacy, and patient enrollment, etc. Blockchain technology can overcome these problems. The following are the few systems providing data integrity and privacy in the clinical healthcare system.

Healthcare is a token-based currency used to record data related to hospital employees, doctors, health plans, and insurance companies, etc. Fast Healthcare Interoperability Resources (FHIRChain) [26] is a smart health system based on smart contracts used to exchange clinical healthcare data. Similarly, Connecting Care [27] is also a blockchain-based record-sharing platform accessed in different cities in England. Connecting Care is used for securing data related to hospitals and other medical record data in a diverse healthcare organization. It provides the access control list, which provides security and allows only authorized users to access the clinical system. In [28] the author presented an Ethereum-based system used to provide smart contract functionality in the blockchain. The system uses an enrollment approach to enroll a patient in the Healthcare system. The medical information of the patient is available to the authorities and allows the patient to contribute his/her personal information to the system. The authors in [29] implemented a blockchain-based secure patient information system that is used to track and store patient data securely, unfalsifiable, and publicly.

### 2.3. Blockchain in the Pharmaceutical Industry/Medical Fraud Detection

In the modern era, every human has a fundamental right to receive health facilities. According to the World Health Organization (WHO), tens of thousands of people die in developing countries due to drug counterfeiting. It is hard to detect fake drugs because these medicines came to the seller through a complex network and it is impossible to track the authentic supply chain. The emergence of blockchain technology has revolutionized the traditional supply chain and can keep track of each party in the supply chain. In [30], the author presented a secure drug supply chain to keep track of each individual in the supply chain to counter drug counterfeiting. The proposed system was developed based on Hyperledger Fabric and allow doctors, patients, nurses, and pharmacist to enter their data in a secure supply chain network. The authors in [31] proposed an improved blockchain platform for the drug supply chain, which uses smart contracts to verify and authenticate the data. The proposed system also provides high transparency and traceability to prevent counterfeiting. Therefore, it is difficult to fabricate data without authorization from the members of the supply chain. In another study [32,33], a digital drug control system (DDCS) was developed based on Hyperledger Fabric, a research project of the Linux Foundation that aims to prevent drug counterfeiting. The system is also responsible for maintaining and supplying authentic drug supply in a drug supply chain. The proposed system also enables administrators to manage patients and doctors. MediLedger [34] is a blockchain-based system using a peer-to-peer paradigm used in the pharmaceutical industry. The designed system shares and validates data across organizations. The permissioned-based system uses private message to exchange data within the network.

The proposed blockchain-based approach provides different types of features, such as better and more effective fault tolerance capability, enhanced system reliability and scalability, and lower operation costs. These features set up a blockchain-based echo system to communicate all registered devices with each other in a secured and distributed manner. The authors proposed a lightweight platform, which maintains most of the security and privacy benefits of blockchain technology for IoT devices to overcome the overheads of classic blockchain models [35].

Gupta et al. [36] presented a model based on unknown inputs with minimal sensing for fractional dynamics. The proposed system processes systems like neurophysiological signals including ECG and $SPo_{2}$. The main contribution of this system is an alternative approach that finds the optimal parameter for the model, retrieves the state of the presented scheme, and is based on optimal parameters and states. They compute a set of recoverable parameters. In another study, Gupta et al. [37] describe an approach for comparing existing ECG-based brain interfacing with a current time-varying sophisticated approach that uses invasive and non-invasive techniques based on machine-learning algorithms. The system accuracy in terms of classification is more involved with having fewer training samples. Moreover, the designed system uses EEG datasets to evaluate the system methodology.

Xue et al. [38] proposed a sophisticated mathematical approach for constructing complex dynamics. The proposed system uses a framework based on casual inference integrated with a probabilistic model to distinguish short- and long-range dynamics dependencies. Moreover, we also use the entropy function for the multi-point probability that differentiates between complex and memoryless time-dependency structures. Similarly, Xue [39] presented a bream machine body interface for a cyber-physical system using the spatiotemporal fractal approach. The developed approach uses a mathematical model for capturing spatiotemporal cross dependencies in terms of coupled processes and brain-machine body interfaces.

Ghorbani et al. [40] presented a mathematical model integrated with a hardware module for artificial pancreas design. The performance of the system is measured by comparing real-world measurements using a conventional nonfractal model. Moreover, we also prove the practicability of in silico realization of the developed optimal control algorithm using a field-programming gate array platform [41].

As aforementioned, these blockchain-based platforms are neither permission-less nor open-source; hence, the general user is unable to upgrade or modify the existing system for their purpose. Moreover, the majority of the methods presented in the literature review are related to managing EMR or sharing the record of doctors, patients, and nurses in the network. Nonetheless, none of any previously presented systems addresses secure vital-sign monitoring using a permissioned blockchain platform known as Hyperledger Fabric. Furthermore, most of the healthcare systems discussed above use an inherent cryptocurrency, which decreases the performance of the system in terms of computational power during the transaction. According to the best of the authors’ knowledge, there has been no functional blockchain-based model developed for the secure and reliable monitoring of patient vital signs based on Hyperledger fabric.

## 3. System Model

**Figure 2:**
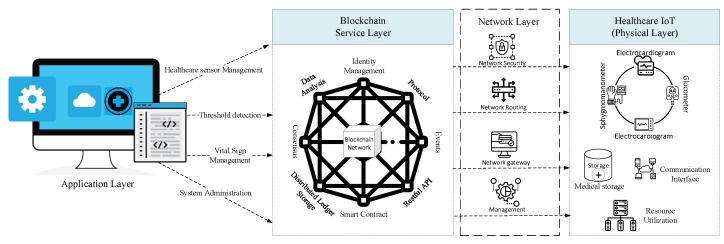
Proposed layered-based healthcare IoT blockchain platform architecture for secure vital-sign monitoring.

The designed healthcare IoT blockchain platform is a modular architecture in which each layer is decoupled from other layers. The decoupled feature enables the developers to add and remove any module without affecting the rest of the system. The developed system is comprised of four layers, i.e., application layer, IoT blockchain service layer, connectivity layer, and healthcare IoT physical layer. The proposed healthcare IoT physical layer comprises various healthcare devices with the capabilities of computing, data storage, and communication. The connectivity layer aims to provide routing management because physical healthcare sensing devices have no global internal protocol. The connectivity layer is also responsible for providing services including security management, message brokers, and network management. Similarly, the IoT blockchain service layer is capable of organizing blockchain-related services that include, e.g., consensus, identity management, distributed ledger technology (DLT), and peer-to-peer communication (P2P), etc. The DLT is a consensus of shared, synchronized, and replicated digital data that is distributed across the entire blockchain network, where every participant has their copy of the ledger. Moreover, the DLT also stores and secures the vital-sign sensing data provided by the healthcare sensor. Any change in the DLT is reflected in all copies across the entire blockchain. The big data module enables blockchain to store data online, which makes blockchain more efficient and reliable. In  blockchain, massive transactional data from different parties are stored in structured forms in ledgers, which is further used in the analysis process. Moreover, all parties in blockchain have granted access to a single network, which makes it easy for the client to access these details. The smart contract is a piece of computer code considered to be a computer protocol triggered by the external client application to manage, access, and modify the ledger. Additionally, a smart contract is also initiated and installed on each peer in the network. Event management in the proposed system is responsible for sending a notification every time a new block is added to the ledger against a predefined condition being satisfied. The application programming interface (API) exposes the developed services provided by the designed medical blockchain platform through which client accesses the application and manages the blockchain network. Blockchain technology allows users to communicate and securely share their resources and assets. Communication in the blockchain is based on a P2P network, consensus algorithms, and asymmetric ciphers. Lastly, the application layer is the top layer, which is a user interface and responsible for vital-sign data visualization and used to manage and control healthcare devices. The proposed layered-based healthcare IoT blockchain platform for secure monitoring of patient vital signs is illustrated in Figure 2.

### 3.1. Interaction Model for the Proposed Healthcare IoT Blockchain Platform

**Figure 3:**
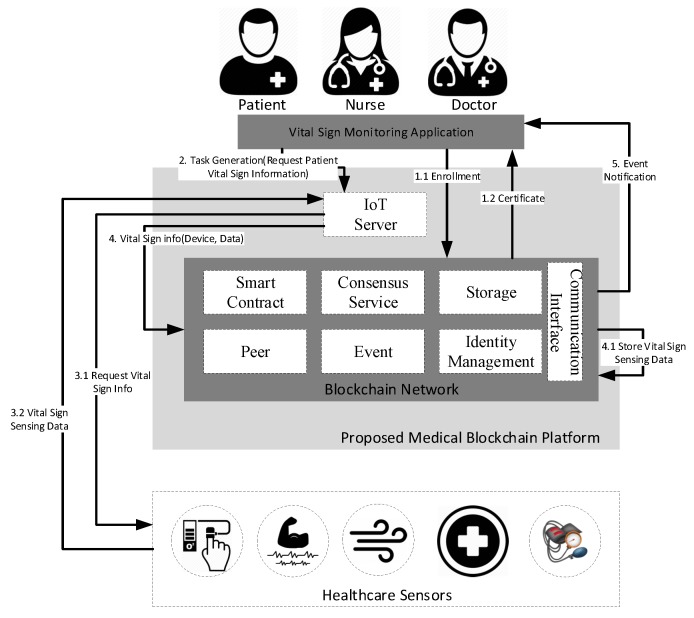
System workflow of the proposed healthcare IoT blockchain platform.

The workflow of the proposed healthcare IoT blockchain model is illustrated in Figure 3. The developed system is comprised of the technical infrastructure that exposes the smart contract and DL through a user service framework as a service to the blockchain. The medical blockchain model comprises a reliable authorized peer, where every individual peer holds the replica of the ledger for the blockchain network to preserve the uniformity of the distributed ledger. The distributed ledger consists of a chain of blocks to store the immutable transactions in the blocks, and a data lake to store and maintain the medical data related to healthcare sensors, and other related participation of the network. The blockchain network is used as transaction logs that record and maintain all the changes that occur in the data lake. The data lake acts as an off-chain ledger (database) used to store the following details of patients, such as the updated values of vital signs and healthcare device information, etc. The off-chain database is further used for data analytics and other healthcare services, e.g.,  critical care, intensive care, and preventive care response. Furthermore, the client application uses the REST API to manage the blockchain network by submitting a transaction request, e.g., task generation service and user and device registration. Every participant is required to enroll in blockchain before submitting their transaction. The enrollment certificate contains a private key that is required to sign the transaction. The transaction in the blockchain network is defined as reading and writing data from the distributed ledger. The participant (i.e., patient, nurse, and doctor) can submit a transaction either to generate a new task or to get a response from the previously generated task through the healthcare IoT server. Afterwards, the healthcare IoT server sends a request to the blockchain network to perform a task according to the request. Furthermore, the healthcare IoT server also transfers tasks generated from the client to fetch real-time vital-sign information, device information, and device status. The gathered information, which includes vital-sign data, device information, and device status, is stored in the ledger along with the specific patient data defined according to the smart contract. Finally, the notification is generated to the concerned participant upon the successful transaction.

### 3.2. Execution of Proposed Healthcare IoT Blockchain Platform

**Figure 4:**
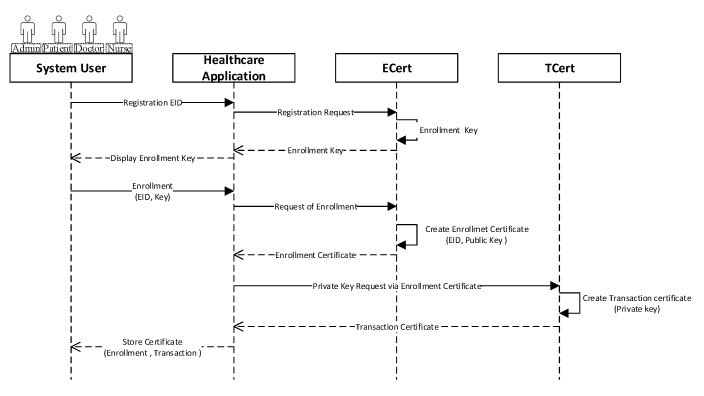
Identity issue for network user.

The execution procedure of the proposed healthcare IoT platform is presented in Figure 4. Generally, in blockchain, before submitting the transaction, every user must have to sign the credentials to be considered an authorized user of the system. Every participant, including a doctor, patient, and nurse, sends the registration request to the blockchain network. The registration request is further received by the identity management, which is responsible for issuing a secret key for authorizing an enrollment process through a client application. Afterwards, the enrollment certificate (ECert) is generated and passes to the client along with the public key for the response. Finally, a transaction certificate (TCert) is generated using ECert, where TCert is further used for signing the transaction.

**Figure 5:**
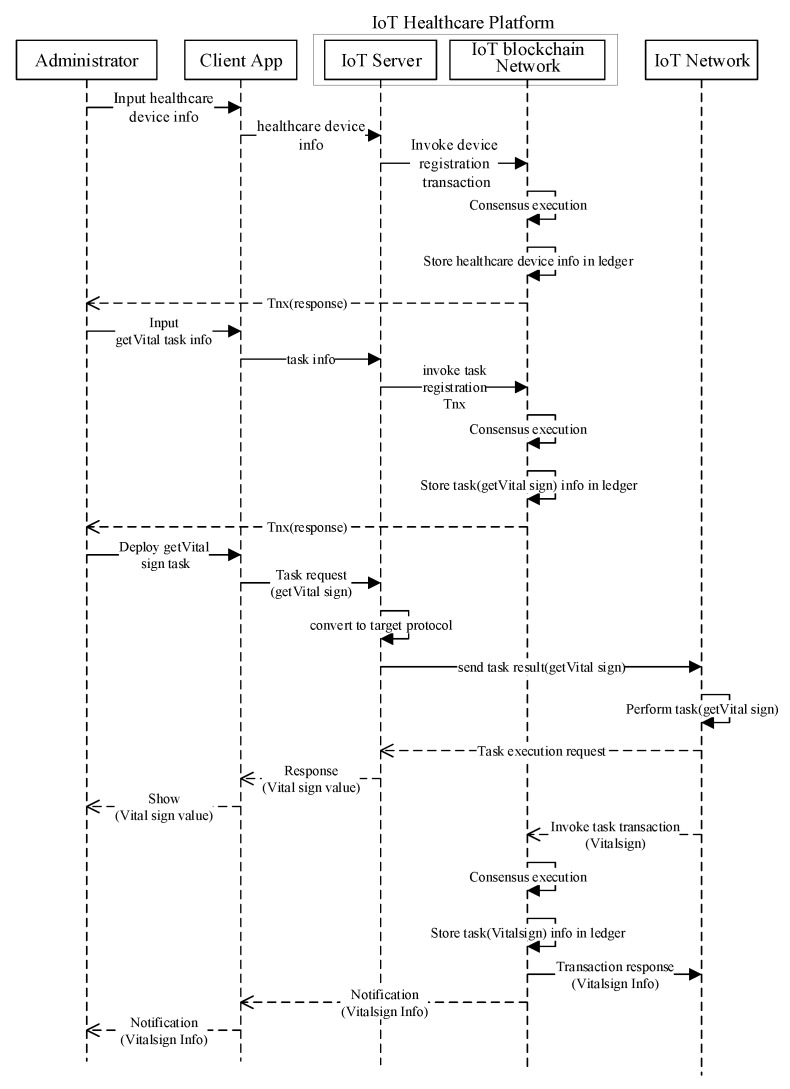
Sequence diagram of operation in the proposed healthcare IoT platform.

After the successful enrollment, only authenticated users can access and consume blockchain services, as visualized in Figure 5. The users use client applications to request information about vital signs. The vital-sign information, along with the request, is sent to the server, which, in return, triggers the smart contract related to that transaction. Afterwards, in the blockchain network, the consensus process is executed, which appends the transaction information in the blockchain and records the vital-sign information in the state database. Finally, the notification is sent to the client regarding the ledger upgrade after the successful execution of the transaction.

Furthermore, users can also generate tasks to perform operations (e.g., read heart rate from the ECG sensor and read airflow rate using a nasal airflow sensor) on healthcare sensors. Moreover, users can also specify certain tasks based on the requirement. The specific task request is sent to the healthcare IoT server, which further translates the request into the defined protocol of the sensor and transfers the request to the specified sensor to perform operations. The target sensor behaves according to the request and returns vital-sign data to the healthcare IoT server as well as to the blockchain network. This vital-sign information in the form of results are displayed to the user in the client application. Moreover, the computed result is also sent as a payload of the transaction to the blockchain network. Finally, the vital-sign information is appended to the distributed ledger of each peer, and it also sends the notification in the case of exceeding the predefined threshold, e.g., in the case of a body temperature sensor the normal range is between 97.7 °F and 99.5 °F.

## 4. Implementation Environment of Proposed System

**Figure 6:**
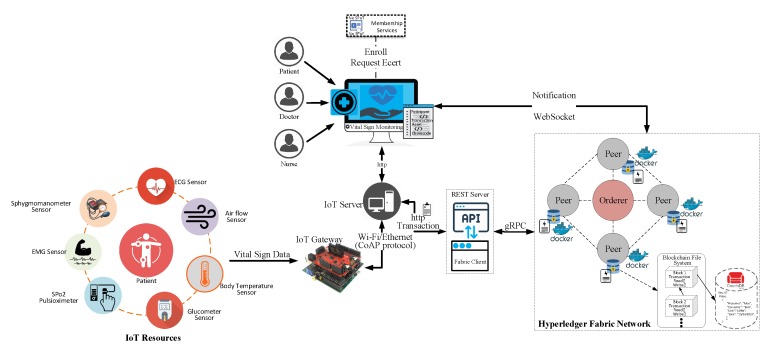
Healthcare IoT blockchain platform implementation and use case deployment for secure monitoring of vital signs.

The implementation environment of the proposed blockchain-based monitoring of the patient’s vital signs is presented in Figure 6. We implemented a case study in which the patient is equipped with healthcare sensors to monitor vital signs using blockchain-based technology on the Hyperledger Fabric framework. Our proposed model aims to establish communication between IoT resources, the healthcare IoT server, and the blockchain network. The IoT devices are healthcare devices, such as ECG sensors, sphygmomanometer sensors, EMG sensors, $SPo_{2}$ sensors, body temperature sensors, etc. A Raspberry Pi is equipped with Libelium e-Health toolkit that acts as an IoT gateway, which routes the vital-sign data to the healthcare IoT server. The healthcare IoT server is responsible for processing requests and providing the required sensors reading to the client through the blockchain network. The processing includes but is not limited to filtering the data, checking whether the vital-sign reading is normal or abnormal, and laying out the data in a format that can be effectively used by the client devices. The proposed system uses the Hyperledger Fabric framework to establish the blockchain network with four peers with an orderer node working as an image in a docker container. Every peer in the proposed blockchain network is comprised of data storage and smart contracts to write transactions to the blockchain ledger. The data storage is a DB couch act as a state database with rich query features, and also supports modeling of a smart contract as JavaScript Object Notation (JSON). Moreover, the Hyperledger composer–rest–server provides the functionality of the Representational State Transfer (REST) Application Programming Interface (API) to expose the services to the client application for further analysis. All the services written in the smart contract can be accessed through REST API using the client application. Additionally, fabric client also uses the Google Remote Procedure Calls (gRPC) in order to communicate with the fabric network. The blockchain network also generated notifications for the client through WebSocket.

### Smart Contract Modeling

This section presents smart contract modeling, including participants, assets, and transaction descriptions defined in the proposed medical blockchain. The smart contract is a computer program intended to enforce custom-designed functionalities in the system and compiled in the form of a business network archive (BNA). In the proposed system, we used Hyperledger composer [42] to design and implement the smart contracts for the secure monitoring of patient vital signs. The Hyperledger Composer is an open-source framework specifically built for developing blockchain-based applications. The smart contract is comprised of four main parts—the model, transaction, query definition, and access control rules. The model file further contains participants and assets. The participants are the users of the system who are responsible for managing and interacting with the system. Similarly, assets are the valuable entities, services, or goods that are used between the participants and stored in blockchain registries.

Furthermore, transactions are logical operations defined in the smart contract that can interact with assets. The transactions are responsible for modifying the value of participants and assets in the blockchain network. The third part is Access Control Language (ACL), which aims to provide authentication and authorization to the participants within the network and also define the roles for each participant in the business network domain model. Furthermore, in ACL, we defined the fourth and last part of the smart contract, which is the queries that are written in a separate file in the bespoke query language. The Hyperledger composer queries are used to retrieve customized data based on user requests from the world state database. Table 1 summarizes the smart contract modeling of the proposed system with participants, assets, and transactions. The participants are doctors, patients, and nurses, whereas the assets are sensors, vital-sign readings, and patient health records. Finally, the transaction processor functions are getSensorReading, addSensor, UpdateSensor, Threshold Detection.

The designed BNA is further used to generate REST API, which is used to interpret the restful services to the client application. The REST API is platform-independent and can be accessed from any platform with valid credentials. The purpose of creating the REST API is to establish communication between the BNA and the front-end client application. The designed REST API is comprised of three sub-parts—resource, verb, and action. The resource is the request URL, whereas the verb is the action, which is going to be performed on a particular resource, such as PUT, POST, GET, and DELETE. Table 2 highlighted the REST APIs produced by the composer–rest–server to communicate between healthcare devices, web clients, and the blockchain network. The REST APIs are based on HTTP protocol and comprised of the following parameters in the header file: media type, verb, and base URI. The verbs are the action performed on the specific resources, such as POST, PUT, GET, and DELETE. Similarly, media type defines state transition elements, e.g., Application/JSON. Lastly, the URI determines the path of the request data entry, for instance, Get request to the resource URI like /api/VitalSignReading would return a list of vital-sign information from a specific healthcare sensor. In contrast, the POST request to the same URI will request the healthcare IoT server to accept the packet encrypted in the URI request.

Algorithm 1 presents the scenario of secure vital-sign sensing from the Libelium e-health sensor based on blockchain. The set Patient, Doctor, and Sensor includes lists of patients, doctors, and healthcare sensors, which is registered in the blockchain network and passed as an input. Similarly, the output is a vital sign of the patient. The process of secure and reliable monitoring of vital signs starts when the user of the system selects from the set of the patient. Once the patient is selected from a set of the patients, then the doctor assigns a healthcare device to a specific patient to get vital-sign information. Afterwards, the vital sign is successfully stored in the blockchain ledger. The getVitalSign function takes a list of patient and healthcare sensors as a parameter. First, the assigned patient and sensor is selected from the list of patients and sensors. The user can map the patient and sensor based on a one-to-one or one-to-many relationship. The vital sign is stored as a JSON array and send healthcare IoT server using a POST request. The communication between the IoT gateway and healthcare IoT server is via the CoAP protocol.
**Algorithm 1:** Securing patient’s vital signs using blockchain**Input**: Patient = {$P_{1}$,$P_{2}$,$P_{3}$,…$P_{x}$} Doctor = {$D_{1}$,$D_{2}$,$D_{3}$,…$D_{y}$} Sensor = {$S_{1}$,$S_{2}$,$S_{3}$,…$S_{z}$} **Output**: $getVitalSign(P_{x},S_{z})$
Successfully stored vital sign in blockchain 
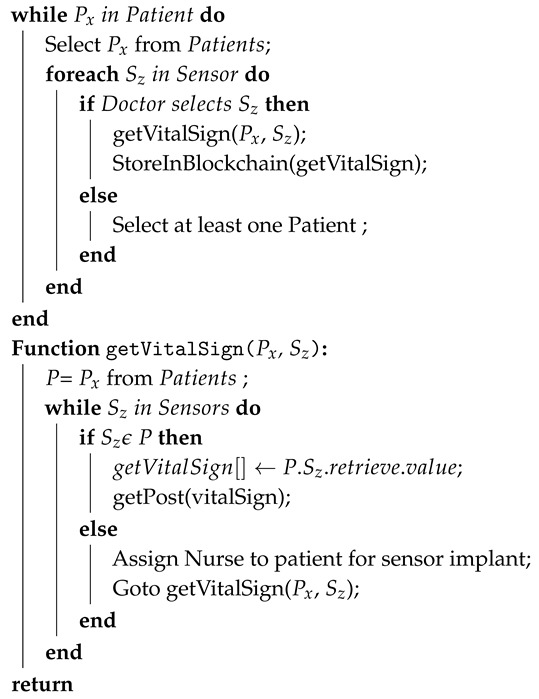


## 5. Development Environment

This section provides an overview of the tool and technologies used in developing the proposed medical blockchain. The development environment of the proposed system is divided into four parts—the development of medical blockchain, Raspberry Pi-based healthcare IoT server, IoT gateway for sensing vital signs from IoT resources, and finally, front-end client application for interacting with the blockchain network. Table 3 summarizes the technology stack for developing all four modules of the proposed medical blockchain. This work used the Libelium e-Health sensor platform v2 for monitoring patient vital signs. The e-health sensor shield provides nine different healthcare sensors, which are used either for monitoring the real-time state of patients or for acquiring vital-sign data for medical diagnosis. Seven different purpose sensors are used in order to evaluate the performance of our proposed system, which includes a sphygmomanometer, electrocardiogram, electromyography, pulse and oxygen in the blood, body temperature, airflow, and glucometer sensor. Moreover, the e-health sensor platform provides an open-source library written in C++ that is used to sense the vital signs from the sensors. Similarly, the operating system we used is Ubuntu Linux 18.04 LTS, which provides the runtime for the docker engine and docker-composer. The docker engine provides the runtime for the docker, whereas the docker-composer is responsible for configuring the docker image and container in the operating system. Likewise, for blockchain development, we have used Hyperledger Fabric version 1.2, a Linux-based open-source framework. The fabric network develops the client software development kit by using Node version 8.11.4. The Hyperledger Fabric also provides the functionality of web-playground for developing smart contracts. Afterwards, a composer command-line tool allows administrators and developers to manage and deploy the chain code. Furthermore, the data transfer from the IoT gateway to the healthcare IoT server is via CoAP protocol, and HTTP is used for blockchain and healthcare IoT server communication.

### Experimental Environment

**Figure 7:**
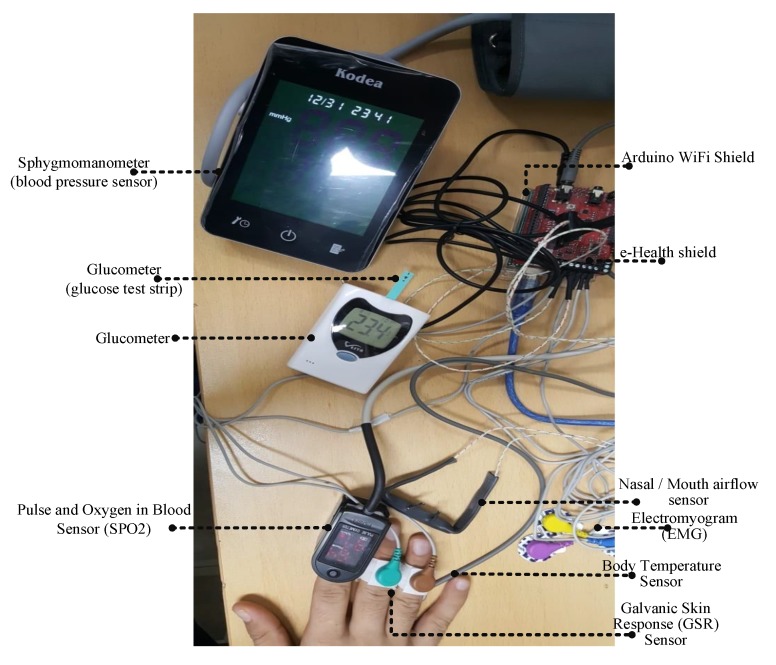
Experimental environment of medical blockchain network.

This section presents the experimental environment of the proposed medical blockchain. In this work, we have designed and developed a testbed for the testing purpose, as shown in Figure 7. We have used a total of 7 healthcare sensors—ECG, EMG, SPo2, glucometer, body temperature, airflow, and sphygmomanometer—in order to monitor patient vital signs. This work proposed a novel medical blockchain-based system where each patient is equipped with healthcare sensors, which can be controlled remotely by doctors and nurses.

Table 4 summarizes the detailed description of healthcare sensors with their normal and abnormal range. In the case of $SPo_{2}$, the normal range should be between 96% to 99%, whereas the abnormal range is below 90%, which may result in headache, restlessness, and other severe symptoms. The $SPo_{2}$ sensor is normally used to measure the amount of oxygen in the blood. Similarly, ECG is used to measure the heart’s electrical activity and rhythm. The normal range of ECG should be up to 120 ms, and the abnormal range is less than 120 ms, which can trigger a medical emergency, such as arrhythmia or myocardial infarction. Airflow or nasal sensor is used to measure breathing rate with the normal range of 15–30 bpm whereas the abnormal range is below 15 and greater than 31. Furthermore, EMG is used to monitor the activity of muscles during contraction, and the normal range of this vital sign is 40 mV–90 mV. Likewise, the sphygmomanometer sensor is used for monitoring blood pressure. All the above-mentioned healthcare sensors are responsible for monitoring patient vital signs. Figure 7 represents the experimental environment of the aforementioned healthcare sensors.

**Figure 8:**
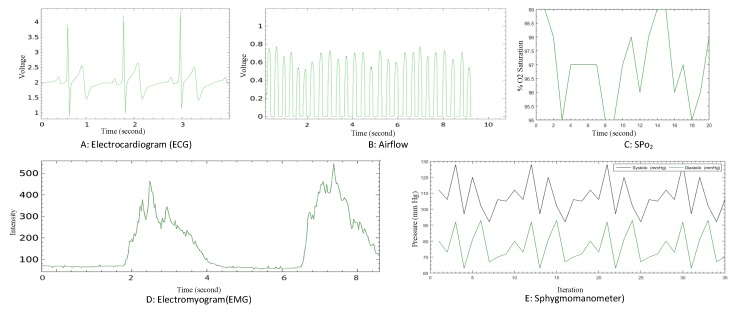
Vital-sign reading with different sensors.

Figure 8 illustrates the visual of vital sign from different healthcare sensors using Libelium e-Health sensor platform. The following five different vital-sign readings are visualized—ECG, airflow, $SPo_{2}$, EMG, and sphygmomanometer, etc. In Figure 8A, we have carried out the electrocardiogram signal of a normal person. The 4 s capturing of ECG from a healthy object shows the normal sinus rhythm where the higher peak represents the rapid depolarization of left and right ventricles. The first interval before the high peak shows the depolarization of the atria, and the second interval visualized the heart rate. Moreover, the first interval was constructed during a relaxed state, and the second interval is plotted while the heart is in a contracted state. Similarly, in Figure 8B shows the pattern of breathing of a healthy object for 10 s. According to the study, a normal person’s respiratory rate is between 15–30 breaths per minute. The short wavelength represents the inhale and exhale of air into the lungs. The crest denotes the inhale, and the trough signifies the exhale deep breath breathing pattern. Likewise, Figure 8C describes the pulse and oxygen in blood using the $SPO_{2}$ sensor. The presented graph shows the amount of $O_{2}$ in the arterial blood of the healthy object over the time duration of 20 s. The normal oxygen saturation in the blood is between 94% to 99% as also visualized in a graph. The electromyogram readings are illustrated in Figure 8D, which is used to analyze muscle rest and contraction activity. Moreover, the signal can also be used to detect health abnormalities, such as neuromuscular, kinesiology, and low-back pain, etc. The high peak between 2–4 s shows the electrical activity while the muscles are in contract state, which considers as normal EMG. The healthy muscle contract rate is between 100 μV to 600 μV. Finally, we also conducted experiments for calculating the pressure of blood in the arteries using a sphygmomanometer sensor. The sphygmomanometer sensor is also known as a blood pressure monitor, which is recorded in two terms, i.e., systolic pressure and diastolic pressure. The systolic pressure is calculated during heartbeats, whereas the diastolic pressure is computed while the heart relaxes between beats. According to the study the blood pressure is classified into six categories, such as hypotension (systolic < 90 and Diastolic < 60), desired (systolic 90–119 and Diastolic 60–79), prehypertension (systolic 120–139 and Diastolic 80–89), stage 1 hypertension(systolic 140–159 and Diastolic 90–99), stage 2 hypertension (systolic 160–179 and Diastolic 100–109), and hypertensive crisis (systolic ≥ 180 and Diastolic ≥ 110). Figure 8E shows the blood pressure of healthy object and prehypertension object. Iteration 9 is an example of a healthy object with the diastolic is 68 mm Hg, and systolic is 94 mmHg. In this work, we carried out multiple tests to check the working of the testbed as presented in Figure 8, and results indicate that the testbed works well on each healthcare sensor used in the proposed medical blockchain.

**Figure 9:**
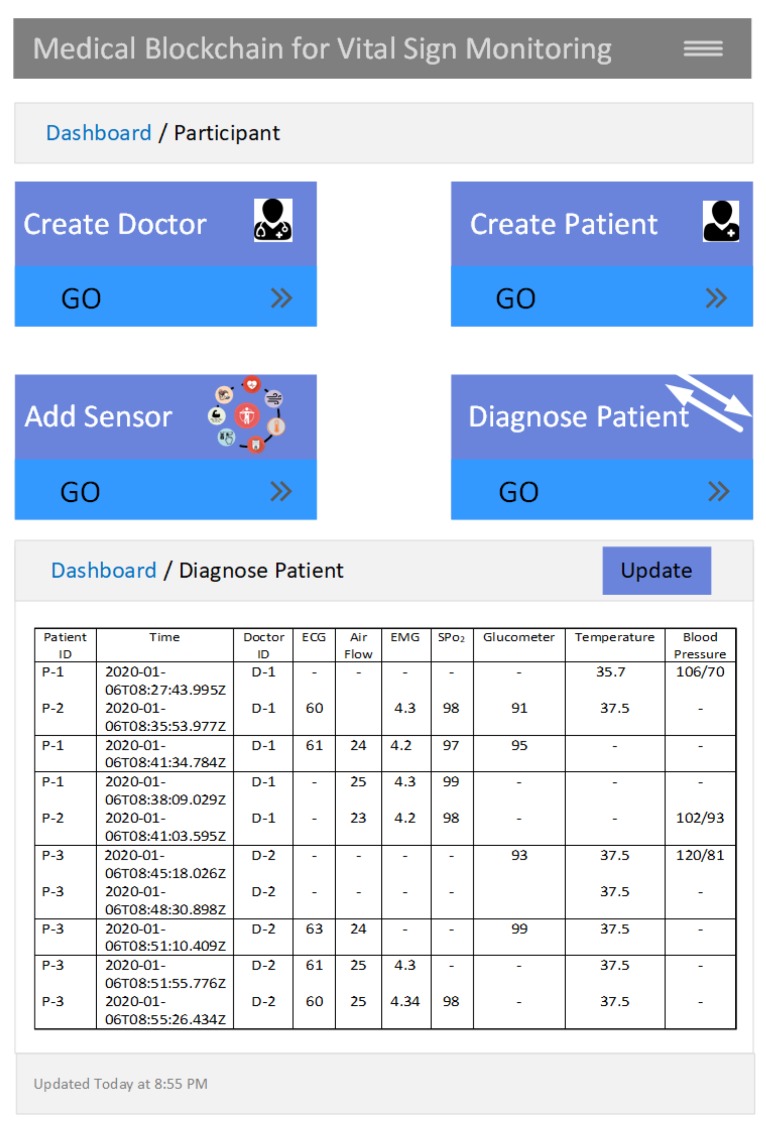
Web-based user interface for vital-sign monitoring with Hyperledger Fabric.

Figure 9 illustrates the web-based user prototype of the medical blockchain implemented using Hyperledger Fabric. The dashboard shows the functionality of the doctor, patient, sensor, and patient vital sign. The Diagnose Patient dashboard shows the records of patientID, DoctorID, along with the vital-sign information. Each record is identified by a unique value of timestamp. The null values indicate that the respective sensor is not installed as per doctor recommendation. The update button updates the value using the composer rest server.

## 6. Performance Evaluation

The performance of the proposed system is evaluated by using Hyperledger Caliper [13], an open-source benchmarking tool for a blockchain platform. The Hyperledger Caliper is developed by the Linux Foundation for measuring the performance of the blockchain-based system. We used Hyperledger Caliper to conduct the performance of the designed system in terms of transactions per second, transaction latency, and resource use. The environmental setup of Hyperledger Caliper is represented in Table 5.

### 6.1. Simulation Results

**Figure 10:**
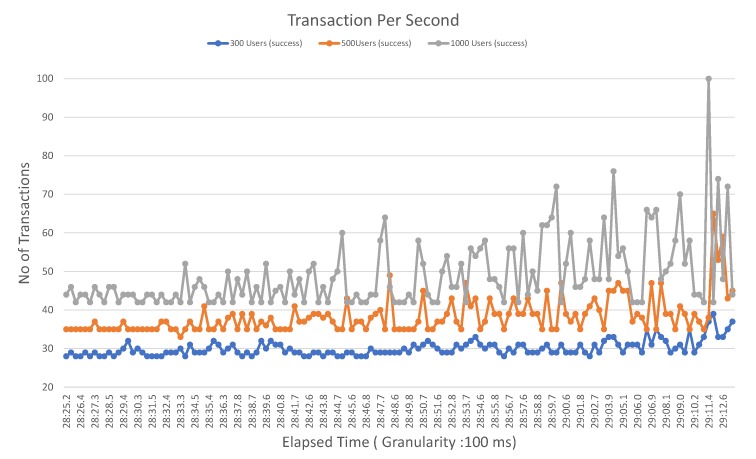
Transaction Per Second (TPS).

In Figure 10, we explain the TPS for the proposed blockchain platform. The TPS is also measured as a throughput. We have taken different user groups to evaluate the performance of the proposed system. The user group is categorized into three categories—300 users, 500 users, and 1000 users. In the first round, we investigate the throughput using 300 users, then in the second round, we take 500 users and lastly with 1000 users. The average throughput suggested that the performance of the system gets enhanced when the number of users is high.

As demonstrated in Figure 10, the average transactions are 30 for the elapsed time of 100 ms for a user group of 300 users. However, if the number of users exceeds 500, the TPS also gets increased with 20 transactions per second. Finally, with 1000 users, the average transactions are 56 TPS.

**Figure 11:**
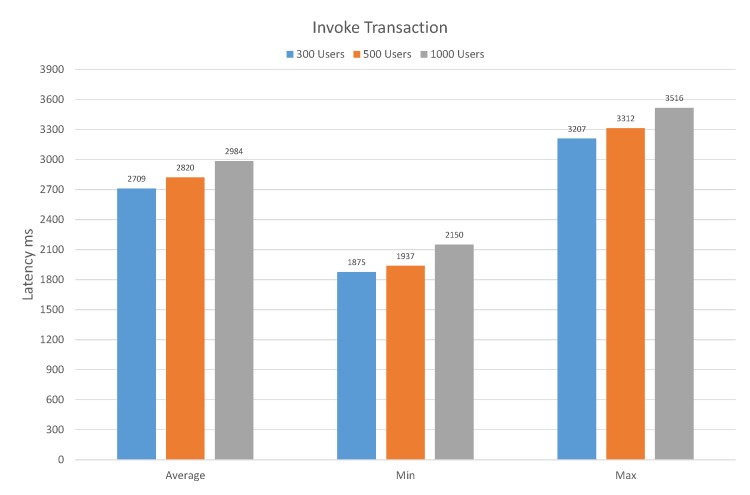
Latency in Invoke Transaction.

In Figure 11, we investigate the latency of executing the invoke transaction of the proposed system with three different user groups in terms of average, minimum, and maximum latency. The user groups are categorized into 300, 500, and 1000 users. If we analyze the user group with 300 users, the average latency is 2709 ms. Likewise, in the case of 500 users, the average latency is increased from 2709 ms to 2820 ms. However, if we increase the user numbers from 500 to 1000, then the average latency is 2984. However, as the number of users increases, the average latency also increases but with a negligible difference. In the case of a user group with 300, 500, and 1000 users, the minimum latency is 1875, 1937, and 2150, respectively. Moreover, the maximum latency in the case of user group with 300, 500, and 1000 are 3207, 3312, and 3516, respectively.

**Figure 12:**
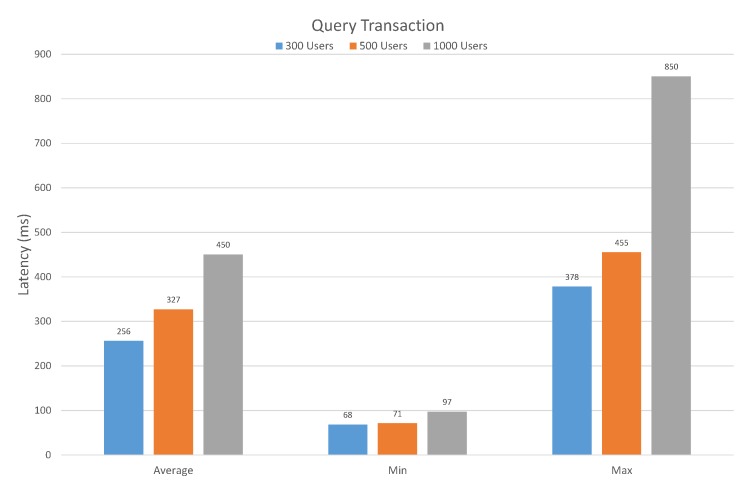
Latency in Query Transaction.

Figure 12 shows the latency of executing the query function with three different user groups. In the case of a user group with 300 users, the average latency of executing query transaction is 256 ms. Likewise, with 500 and 1000 users, the average latency is 327 ms and 450 ms, respectively. Similarly, if we analyze the minimum latency with 300, 500, and 1000 users, the estimated minimum latency is 68, 71, and 97, respectively. However, the maximum latency of executing query transaction with 300, 500, and 1000 users is 378 ms, 455 ms, and 850 ms, respectively.

The performance of the proposed system is also measured using resource use in terms of CPU consumption, memory use, and In/Out traffic. Table 6 summarizes the resource use in terms of CPU (max. and avg.), Response Time Over Time, Memory (avg, max), and Traffic (In or Out). We executed the simulation for the 20 iterations to evaluate the performance of the resource use of the proposed system.

### 6.2. Comparative Analysis

This paper presents a real-life case study for a smart medical solution in smart hospitals, which was developed as a part of the experimental test carried to assess the practicability of the proposed medical blockchain system. The designed medical blockchain platform is based on a permissioned network, with low latency, friendly interface, high transaction throughput, with no currency exchange.

A comparative analysis of the proposed work with some of the similar projects reviewed in the related work is presented. We have conducted a critical review of different state-of-the-art methods in reference to the proposed work. A benchmark study has been conducted to investigate the capabilities of the proposed work in terms of efficiency. The outcome of the benchmark study is shown in Table 7. The parameters which have a leading impact on the system performance have been considered, and the output reflects a significant boost with respect to the existing state-of-the-art methods. The existing approaches, such as MedRed, Robomed Network, and Mediblock, have much more energy consumption and are less efficient because they are permission-less blockchain and need to build consensus among all nodes. Furthermore, the power consumption of these approaches is also on the higher side due to costly mining and smart contract execution. Nonetheless, we have developed the proposed system using a permissioned network, and thus reduces the overall overhead of the system. On the contrary, the proposed medical platform is designed on a permissioned blockchain network, which significantly reduced the overhead while deploying on the network. Nevertheless, many of these systems are totally based on EMR functionality. However, the proposed system not only preserves the electronic medical record but also provides the monitoring of physiological parameters for the various disease through distributed ledger technology. Furthermore, none of the existing medical systems support resource-constrained IoT devices. However, the proposed system provides a lightweight solution that avoids integration blockchain technology into Libelium IoT devices, and these IoT devices do not require any changes. The blockchain in the proposed system is used to provide secure and reliable storage; therefore, the transaction made by the healthcare sensors provided by Libelium is validated by the blockchain network without copying the entire blockchain network. The designed platform improves system usability in many healthcare scenarios with waste capabilities. Furthermore, the webserver API is used to communicate between IoT devices and the blockchain, which provides access to cross-platform communication. The designed system is built on a modular architecture that can be easily extended to meet the requirements of other various domains such as supply chain, renewable energy trading, and IoT-based application.

**Figure 13:**
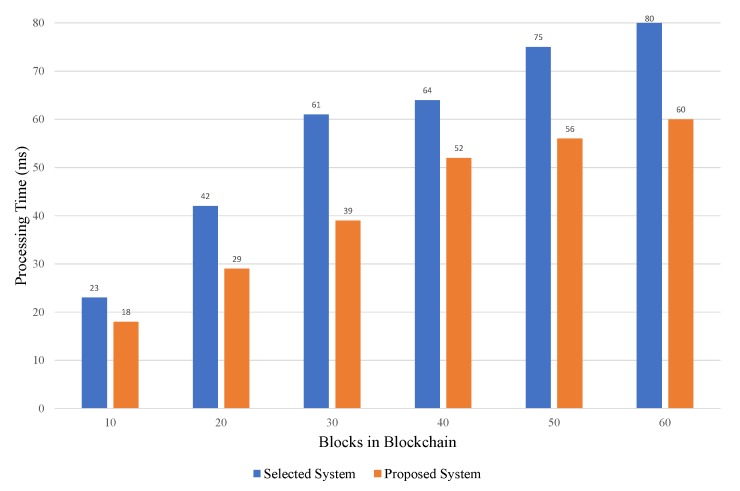
Comparison of processing overhead in proposed healthcare IoT blockchain.

The following properties are considered in order to compare the proposed model with the existing state-of-the-art techniques. These properties play a vital role to demonstrate the effectiveness and significance of our proposed blockchain-based approach. As the comparative analysis of different studies shows in Table 7, it can be observed that the model presented in [35] is the most similar properties or characteristics to our proposed model. Therefore, we chose the most similar model in order to compare with our proposed blockchain-based model to highlight the effectiveness and overall performance of our model. In this comparative analysis, we used a similar simulation environment as used by the selected study [35]. We executed a simulation network of 50 peers for 60 seconds. During the time interval of the 60 seconds, 960 transactions were successfully executed. The processing time metric indicates the time cost in ms to verify a new block in the network. The following Figure 13 presents the simulation results for evaluating the performance of both systems in terms of processing overhead. The processing overhead is an indirect or excess memory, computation time, bandwidth, and other resources that are required for validating a new block in the blockchain network. Based on comparative analysis results, it is found that the processing overhead of our proposed model is lower than the selected model. It can also be observed that the number of blocks in the network vary from 10 to 60 blocks. Hence, our proposed healthcare IoT blockchain model performed well and minimized the processing time by up to 21%.

### 6.3. Featured Application and Challenges

In this paper, we employed a blockchain application prototype to validate the feasibility and usability of the proposed healthcare IoT blockchain solution. The proposed system model can be further protracted to many other useful scenarios like supply chain management, home automation, and the manufacturing industry, etc. The importance of this work can revolutionize the existing shipping and manufacturing industry. Numerous sensors can be plugged into or connected with to different types of goods that are secured and trusted to third-party logistics for transport with trackable history records. The trackable history record includes location, temperature, and humidity data that are easily accessible to the registered users in the network. Similarly, the IoT devices are monitored by a smart contract, which gives a response according to the access control rules specified in the smart contract. In the supply chain, based on the smart contract, the system sends a notification to the staff if the value goes into an abnormal state.

There are some limitations to the proposed system that should be addressed in future work. The main issue is the lack of authentication and the communication between the REST server and the IoT devices, and that may create security issues. Moreover, the health device can invoke the REST API when the composer–REST–server should be configured with different validation strategies. The proposed blockchain system is fully fault-tolerant and reliable, but in case of healthcare IoT network, the IoT server must be configured in a way that it will detect node failure. In the future, we will configure the IoT server in such a way that it frequently sends a packet to the IoT resources, and it will create synchronization between the IoT resources and server. Lastly, for ease of implementation, we deployed the proposed system in a local area network. In the future, we will deploy the proposed method in the cloud environment based on the real-world application requirements.

## 7. Conclusions and Future Direction

The provisioning of a quality health facility is vital for humans and is considered the most significant measure for the quality of lives in a country. The vital signs of humans count the most for their health, and thus physiological parameters for various diseases have been predicted with vital signs. There have been significant breakthrough efforts encouraging the use of blockchain technology to not only provide security but also allow billions of IoT devices aimed for a safe, transparent, and meaningful medical assistance for patients and healthcare providers. This work proposes a novel platform for a decentralized healthcare IoT. The proposed platform is based on permissioned blockchain network and addresses the inherent challenges such as data security, identity, and scalability, to name a few. As part of the implementation, a proof of concept (PoC) is designed. The PoC application has patients equipped with the Libelium e-Health platform to monitor their vital signs. Additionally, the proposed architecture establishes communication among healthcare IoT nodes, the healthcare IoT server, and the blockchain network. Finally, a web front-end application is designed to interact with the blockchain platform for exposing the services to the users. We used Hyperledger Caliper for assessing the performance of the proposed work in terms of various metrics. The performance evaluation suggests that the introduction of blockchain technology not only allows a significant increase in the overall throughput but also reduces the latency. The possible future directions of this work can be the potential to extensively test the interoperability of the proposed system with different IoT frameworks. Also, other consensus algorithms and data storage technologies can be taken into consideration to investigate the improvements in the transaction processing rate and make data queries more efficient.

## Figures and Tables

**Table 1:** Smart Contract Modeling for Proposed Vital-Sign Monitoring.

Type	Component	Description
Asset	Sensor	The sensor asset represents the health sensor, (e.g., ECG, EMG, and air flow sensor etc.) with detailed information like name, sensorID, event_threshold, and unit etc.
	Vital_Sign Reading	The vital-sign reading is the sensing reading of healthcare sensors.
	HealthRecord	The HealthRecord describe the health status of each patient that are equipped with healthcare devices.
Participant	Doctor	User of the system.
	Patient	User of the system.
	Nurse	User of the system.
Transaction	getSensorReading	Transaction which is used to collect vital-sign reading from the healthcare sensors.
	Add Sensor	Add new sensor in medical blockchain
	Update Sensor	Transaction that is used to update the sensor configuration.
	Threshold Detection	Transaction that is responsible for checking whether vital sign is abnormal or normal.

**Table 2:** RESTful API for proposed healthcare IoT blockchain platform.

Operations	Action	URI
Patient Management	ALL	/api/Patient
Doctor Management	ALL	/api/Doctor
Nurse Management	ALL	/api/Nurse
Task Management	ALL	/api/Task
Healthcare Device Management	ALL	/api/Sensor
Retrieve Vital-Sign Data log, Add Vital-Sign Data	GET, POST	/api/VitalSignReading
EMR Management	ALL	/api/PatientRecord
Share EMR record with doctor and Nurse	POST	/api/ShareRecord
Blockchain Network Text	GET	/api/system/ping
Issue an identity to specific participant	POST	/api/SystemIdentities/issue
Retrieve all identities	GET	/api/System/identities
Get all historian records	GET	/api/System/historian

**Table 3:** Development environment for the proposed patient vital-sign monitoring.

Name	Component	Description
Healthcare Medical Blockchain Network	CPU	Intel(R) Core(TM) i5-8500 CPU @3.00 CHz
	Operating System	Ubuntu Linux 18.04 LTS
	Docker Engine	Version 18.06.1-ce
	Docker-Composer	Version 1.13.0
	IDE	Composer Playground
	Programming Language	Node.js
	Hyperledger Fabric	Version 1.2
	Node	Version 8.11.4
	Database	Couch DB
	Memory	12 GB
Healthcare IoT Server	Hardware	Arduino Uno
	Server	CoAP Server
	Library/Framework	Californium CoAP, Http URL Connection
	Programming Language	Arduino
	Operating System	Ubuntu Linux 18.04 LTS
IoT Gateway	Healthcare Toolkit	libelium e-Health Sensor Platform V2.0
	Hardware	Arduino Uno
	Server	CoAP Server
	Library/Framework	Californium CoAP, Http URL Connection
Healthcare Blockchain Web application	Operating System	Window 10
	Browser	Chrome, Firefox, IE
	Programming Language	HTML, CSS, JavaScript, Node.js
	Library/Framework	Notify.js, Californium CoAP, JQuery, Bootstrap

**Table 4:** Details of healthcare sensor used in experimental setup.

Sensor	Model	Abnormal	Normal Range	Description
Pulse and Oxygen in Blood ($SPo_{2}$) [43]	Libelium e-Health Sensor Platform (V-2)	x<90	96–99% 70–100 bpm	$SPo_{2}$ is used to calculate the amount of oxygen in blood and also the tactile arterial palpation.
Electrocardiogram (ECG) [44]		0≤x>120	120 ≤ x > 0	ECG is used to generate electrocardiogram of heart activity.
Airflow [45]		15<x≥31	15–30 bpm	Nasal or mouth airflow is used to calculate the breathing rate.
Body temperature [46]		>40.0–41.5 °C (104–106.7 °F)	36.5–37.5 ° C (97.7–99.5 °F)	Body temperature sensor is used to check the temperature of body.
Sphygmomanometer [47]		Systolic: 90<x>120 (mm Hg) Diastolic: 60>x<80 (mm Hg)	Systolic: 90–119 (mm Hg) Diastolic: 60–79 (mm Hg)	Sphygmomanometer is used to monitor the blood pressure.
Glucometer [48]		100 to 125 mg/dL (5.6 to 6.9 mmol/L)	70 and 100 mg/dL (3.9 and 5.6 mmol/L)	Glucometer is used to estimate the amount of glucose in blood. It is measured in mg/dL or mmol/L.
Electromyogram (EMG) [49]		450 and 780 mV	40 mV–90 mV	EMG measures the electrical activity of muscles atrest and during contraction

**Table 5:** Environmental Setup of Hyperledger Caliper.

Component	Description
Docker Engine	Version 18.06 -ce
CLI Tool	Node-gyp
Docker-Composer	Version 1.130
Node	v8.11.4

**Table 6:** Resource Use Analysis of Proposed System.

Type	Name	CPU	CPU	Memory	Memory	Traffic	Traffic
		(max %)	(avg %)	(max)	(avg)	In	Out
Process	local-client.js	14.64	8.76	105.2 MB	90.5 MB	-	-
Docker	peer1.pump1.com	12.44	5.59	106.6 MB	98.5 MB	1 MB	421.3 KB
Docker	peer0.pump2.com	17.09	6.24	105.7 MB	96.7 MB	1.6 MB	666.7 KB
Docker	peer0.pump1.com	15.02	4.56	89.5 MB	82.3 MB	697 KB	287.6 KB
Docker	peer1.pump2.com	0.00	6.54	112.8 MB	105.8 MB	819 B	0 B
Docker	orderer.com	14.95	6.75	93.6 MB	88.7 MB	5 MB	5.6 MB
Docker	ca_nodeDepartment1	0.00	0.00	5.5 MB	5.5 MB	546 B	0 B
Docker	ca_nodeDepartment2	0.00	0.00	0 B	0 B	0 B	-

**Table 7:** Comparative analysis with existing systems.

Name	Crypto-Currency	Efficiency	Smart Contract	Consensus Determination	Access Policy	Mining Required	Functionality
MedRec [16]	Yes	Low	Yes	Complete Nodes	Premissionless	Yes	EMR management
MediBloc [24]	Yes	Low	Yes	Complete Nodes	Premissionless	Yes	EMR management
Healthcoin [25]	Yes	Low	Yes	Complete Nodes	Premissionless	Yes	EMR management
FHIRChain [26]	No	High	Yes	Single organization	Permissioned	No	EMR management
ConnectingCare [27]	Yes	High	Yes	Single organization	Premissionless	Yes	EMR management
DrugDelivery [30]	No	High	Yes	Selected nodes	Premissioned	No	Drug Management,
MediLidger [34]	No	High	Yes	Single organization	Premissioned	No	Drug Management
IoT Blockchain [35]	No	High	Yes	Arbitrary Nodes	Permissioned	No	IoT Smart Home
**Proposed Platform**	**No**	**High**	**Yes**	**Arbitrary Nodes**	**Permissioned**	**No**	**Vital-sign monitoring, EMR management,** **Medical data preservation, etc.**
